# Effects of *MACC1* polymorphisms on hepatocellular carcinoma development and clinical characteristics

**DOI:** 10.7150/jca.38856

**Published:** 2020-01-14

**Authors:** Chien-Hua Lin, Ming-Ju Hsieh, Hsiang-Lin Lee, Shun-Fa Yang, Shih-Chi Su, Wei-Jiunn Lee, Ying-Erh Chou

**Affiliations:** 1 Department of Surgery, Chang Bing Show Chwan Memorial Hospital, Changhua, Taiwan; 2 Institute of Medicine, Chung Shan Medical University, Taichung, Taiwan; 3 Cancer Research Center, Changhua Christian Hospital, Changhua, Taiwan; 4 Graduate Institute of Biomedical Sciences, China Medical University, Taichung, Taiwan; 5 Department of Surgery, Chung Shan Medical University Hospital, Taichung, Taiwan; 6 School of Medicine, Chung Shan Medical University, Taichung, Taiwan; 7 Department of Medical Research, Chung Shan Medical University Hospital, Taichung, Taiwan; 8 Whole-Genome Research Core Laboratory of Human Diseases, Chang Gung Memorial Hospital, Keelung, Taiwan; 9 Department of Dermatology, Drug Hypersensitivity Clinical and Research Center, Chang Gung Memorial Hospital, Linkou, Taiwan; 10 Department of Urology, School of Medicine, College of Medicine, Taipei Medical University, Taipei, Taiwan; 11 Department of Medical Education and Research, Wan Fang Hospital, Taipei Medical University, Taipei, Taiwan

**Keywords:** Hepatocellular carcinoma, polymorphism, MACC1

## Abstract

Hepatocellular carcinoma (HCC) is a major malignancy of cancer-related mortality worldwide. Metastasis-associated in colon cancer-1 (MACC1) was suggested as a marker for vascular invasive HCC. This study investigated the *MACC1* single-nucleotide polymorphisms (SNPs) to evaluate HCC susceptibility and clinicopathological characteristics. In this study, real-time polymerase chain reaction was applied to analyze five SNPs of *MACC1* rs1990172, rs975263, rs3095007, rs4721888, and rs3735615 in 378 patients with HCC and 1199 cancer-free controls. The results showed that in 151 HCC patients among smokers who carried *MACC1* rs1990172 "CA + AA" variants had a lower risk of developing a large tumor (odds ratio [OR] = 0.375, *p* = 0.026), more advanced clinical stage ([OR] = 0.390, *p*=0.032), and vascular invasion ([OR] = 0.198, *p* = 0.034). In 137 HCC patients among drinkers who carried *MACC1* rs4721888 "GC + CC" variants had a higher risk to develop vascular invasion ([OR] = 3.780, *p* = 0.009). Further analyses revealed a statistical significance of aberrant AST/ALT ratio in HCC patients with *MACC1* rs975263 "AG+GG" variants before adjustment of age and alcohol drinking. In conclusion, our results suggested that the *MACC1* SNPs rs1990172, rs4721888, and rs975263 are involved in HCC progression and clinical characteristics. *MACC1* polymorphisms may serve as a marker or a predictor to evaluate HCC progression and prognosis.

## Introduction

Hepatocellular carcinoma (HCC), a major malignancy of cancer-related mortality in patients with cirrhosis, ranked the third leading cause of cancer death with an elevating prevalence worldwide 1, 2. Risk factors such as age, alcohol drinking, hepatitis-B virus (HBV), cigarette smoking, sex, severity of cirrhosis were suggested to be correlated with the risk of HCC and disease progression 3-8. In Taiwan, HCC is responsible for the first or second highest cause of cancer death even though a nation-wide infant hepatitis B vaccination program was performed in last two decades 1, 9-12.

Metastasis-associated in colon cancer-1 (MACC1) was first identified in colon cancer and was suggested as an oncogene 13-15. In HCC, MACC1 was suggested as a marker for vascular invasive HCC 16, 17, and high intratumoral MACC1 expression was suggested to be correlated with increased tumor progression and poor outcome of hepatitis B virus-related HCC 17, 18. Moreover, the *MACC1* gene was identified to play essential role in regulating c-MET proto-oncogene expression (c-MET) and was suggested as a novel prognostic marker for HCC 19-21.

The MACC1 expression or its functions may be altered by its single nucleotide polymorphisms (SNPs), which consequently influence the progression of cancer development. Revealing studies have identified the association of *MACC1* SNPs expression to clinical outcomes and cancer prognosis 22-26. In HCC, SNPs of *MACC1* was suggested to be potential genetic markers for HCC recurrence for those patients who received liver transplantation (LT) 27. However, the exact role of *MACC1* SNPs in Taiwanese HCC patients to cancer progression and development remained not well-investigated. In the current study, we selected five *MACC1* SNPs rs1990172, rs975263 (exon 5), rs3095007, rs4721888 (exon 4), rs3725615 (exon 7), and try to elucidate their correlations to Taiwanese HCC patients and cancer prognosis.

## Materials and Methods

### Study subjects

For the study group, we consecutively recruited 378 patients including 266 men and 112 women during 2007-2015 at Chung Shan Medical University Hospital in Taichung, Taiwan. For the control group, we selected 1199 cancer-free controls including 839 men and 360 women from the Taiwan Biobank. The patients and the normal controls with any histories of other cancers were excluded, and we enrolled the patients with only HCC in our study. The information and exposure to risk factors such as alcohol drinking, cigarette smoking was administrated with a questionnaire for both the controls and the study group, and we classified the history of exposure into "ever user" or "never user". The medical information of HCC such as TNM clinical staging, tumor size, lymph node metastasis, distant metastasis, Child-Pugh grade, vascular invasion, HBsAg, Anti-HCV and liver cirrhosis to those individuals enrolled in our study were obtained from their medical records. Written informed consent was acquired from each participant enrolled in this study. The approval of this study was approved by the Institutional Review Board of Chung Shan Medical University Hospital (CS17132).

### Sample preparation and DNA extraction

The peripheral blood specimens from HCC patients and normal controls were collected for genomic DNA extraction. The whole blood samples were placed in EDTA containing tubes and were centrifuged at 3000 rpm, 10 minutes as soon as possible. The genomic DNA extraction was performed with QIAamp DNA blood mini kits. The buffy coats extracted from the whole blood specimens were used for DNA extraction, and the DNA extraction assay was performed according to previous described 28-30. Extracted DNA was dissolved in Tris-EDTA (TE) buffer and was applied as DNA template in the following process of polymerase chain reactions (PCRs).

### Selection of *MACC1* SNPs

A total of five SNPs rs1990172, rs975263, rs3095007, rs4721888, and rs3735615 in *MACC1* were selected from the International HapMap Project database for our current study 31. The SNP rs1990172 was selected because this SNP was suggested as a predictor to evaluate reduced overall survival in colorectal cancer patients 22. The *MACC1* rs3735615, rs4721888 and rs975263 were selected because these SNPs were suggested to be associated with the risk of breast cancer susceptibility 24, 25. The rs3095007 was selected because it is one of the common variants representing the majority of *MACC1* locus 32.

### Statistical analysis

To compare the age, gender, cigarette smoking, alcohol drinking, HBsAg, anti-HCV, tumor stage, tumor T status, lymph node status, metastasis, Child-Pugh grade, and liver cirrhosis between the healthy controls and patients with HCC, Mann-Whitney U test or Fisher's exact test was used. p < 0.05 was considered that a significant does exist. Logistic regression models were used to estimate the odds ratio and 95% CIs of the association between the genotype frequencies and HCC risk and the clinical pathological characteristics. All of the data in the current study were analyzed on SAS statistical software (Version 9.1, 2005; SAS Institute, Cary, NC).

## Results

Table 1 presents the distribution of demographic characteristics in 1199 controls and 378 patients with HCC. After we analyzed these demographic characteristics, we observed that 14.1% (169/1199) of the controls and 36.2% (137/378) of the patients with HCC drank alcohol. Significant distributional differences were observed for age (p < 0.001), and alcohol drinking (p < 0.001) between the controls and patients with HCC.

The genotyping and allele frequency of *MACC1* SNPs in the patients with HCC and healthy controls are shown in Table 2. The highest distribution frequencies in the controls and patients with HCC of *MACC1* genetic polymorphisms rs1990172, rs975263, rs3095007, rs4721888, and rs3735615 were homozygous for CC, homozygous for AA, homozygous for CC, homozygous for GG, and homozygous for GG, respectively. In our recruited control group, the frequencies of *MACC1* SNPs were in Hardy-Weinberg equilibrium. After adjustment for the effects of age and alcohol drinking, no significant differences were observed for the patients with HCC among the rs1990172, rs975263, rs3095007, rs4721888, and rs3735615 polymorphisms of the *MACC1* gene and those with the wild-type (WT) gene (Table 2).

To clarify the role of *MACC1* genetic polymorphisms in HCC status in relation to clinical stage, tumor size, lymph node metastasis, distant metastasis, vascular invasion, Child-Pugh grade, HBsAg, anti-HCV, and liver cirrhosis, the distribution frequency of clinical status and *MACC1* genotype frequency in the patients with HCC was estimated. The rs975263, rs3095007, and rs3735615 genetic polymorphisms showed no significant association with clinicopathologic status (data not shown). However, we found that in 151 HCC patients among smokers who carried the polymorphic rs1990172 gene had a lower risk of clinical stage (odds ratio [OR] = 0.390, 95% confidence interval [CI] = 0.165-0.924, p = 0.032), tumor size (OR = 0.375, 95% CI = 0.159-0.888, p = 0.026), and vascular invasion (OR = 0.198, 95% CI = 0.044-0.882, p = 0.034) than did those carrying the rs1990172 WT gene, but no differences were observed for lymph node metastasis, distant metastasis, Child-Pugh grade, HBsAg, anti-HCV, and liver cirrhosis (Table 3). An opposite result was observed in 137 HCC patients among drinkers who carried the polymorphic rs4721888 gene, who had a higher risk of vascular invasion (OR = 3.780, 95% CI = 1.396-10.230, p = 0.009). However, no differences were observed for other clinical statuses (Table 4).

To further analyze and elucidate the relationship between the level of clinical pathological markers and progress of clinical status in patients with HCC, we analyzed the levels of common clinical pathological markers of HCC including α-fetoprotein (AFP), aspartate aminotransferase (AST), and alanine aminotransferase (ALT) associated with MACC1 genotypic frequencies. Table 5 presents the associations of MACC1 genotypic frequencies with HCC laboratory status. After adjustment for age and alcohol drinking, a significant association was observed between the *MACC1* rs975263 polymorphism and AST/ALT ratio (p = 0.021). However, no significant association was found between MACC1 rs1990172, rs3095007, rs4721888, rs3735615 polymorphisms and HCC laboratory findings (Table 5).

## Discussion

In this study, we demonstrated the correlations between *MACC1* SNPs and HCC. Alcohol drinking is a well-established risk factor for liver cancer 33, 34, and other risk factors such as cigarette smoking, age, sex, severity of cirrhosis, HBV, and diabetes were suggested to be suspected or potential candidates to influence the risk of HCC 4, 5, 7, 8. In our study, the statistical significant association of demographical characteristics between controls and patients with HCC was found in age (*p* < 0.001) and alcohol drinking (*p* < 0.001), but not cigarette smoking (*p* = 0.818) and gender (*p* = 0.884). In a study of non-small cell lung cancer (NSCLC), MACC1 and c-met were associated with poor prognosis in patients with NSCLC, and MACC1 was suggested as an independent prognostic factor for NSCLC, but there is no significant association with sex, age, smoking, and histological classification between MACC1 and c-met expressions 6. Compared with this result, although the information and relationship of carcinogenic risk factor exposure to MACC1 expression is limited, it seemed that the interaction and influence between *MACC1* and risk factors varies in different cancers.

We further analyzed the genotype distributions of *MACC1* gene polymorphisms in 1199 controls and 378 patients with HCC. In colorectal cancer, *MACC1* SNP rs1990172 was suggested as a predictor for reduced overall survival in colorectal cancer patients 22. The CT genotype of *MACC1* rs975263 was suggested to be associated with a reduced survival for younger colon cancer patients in early stages 23, and TT genotype of SNP rs1990172 in gene *MACC1* was associated with worse disease-free survival (DFS) in patients of resectable colorectal cancer (CRC) and was found to exhibit higher frequency in patients with T3/T4 staging 26. In patients with human epidermal growth factor 2 (HER2)-positive breast cancer, the *MACC1* SNPs rs1990172, rs975263, and rs3735615 were associated with clinical outcome such as increased risk for progression or death and a significant protective impact on event-free survival and overall survival 24. Moreover, SNPs rs1990172 and rs975263 in the *MACC1* gene was suggested to play a role as potential genetic markers for HCC recurrence in patients undergoing liver transplantation (LT) 27. However, compared with these results, we found that there is no significant association of *MACC1* SNPs rs1990172, rs975263, rs3095007, rs4721888, and rs3735615 between HCC patients and normal controls in our study (Table 2). This result implied that the direct impact of *MACC1* polymorphisms on HCC carcinogenesis may be limited in Taiwanese HCC population. Since the *MACC1* gene was found to play a role in promoting tumour cell growth and the development of distant metastasis through upregulation of c-MET 19, and *MACC1* was suggested to serve as a possible biomarker in HCC 21. Therefore, the *MACC1* SNPs expression may play a more essential role and key regulator in HCC progression rather than HCC carcinogenesis.

We further analyzed the correlations of *MACC1* SNPs expression and clinical status in HCC patients. Although there is no significant difference of cigarette smoking between the control group and patients with HCC in our study (*p* = 0.818; Table 1). However, in HCC patients among smokers, we found that carriers of the *MACC1* rs1990172 "CA+AA" genotypic variants revealed a lower risk in clinical stage ([OR] = 0.390, *p* = 0.032), tumor size ([OR] = 0.375, *p* = 0.026), and vascular invasion ([OR] = 0.198, *p* = 0.034) compared with the *MACC1* rs1990172 "CC" genotypic variant (Table 3). In contrast, a significant association was found in alcohol drinking between the HCC patients and controls (*p* < 0.001; Table 1), and we found that carriers of *MACC1* rs4721888 "GG" and "GC+CC" genotype in HCC patients among drinkers have higher risk to develop vascular invasion ([OR] = 3.780, *p* = 0.009) compared with the *MACC1* rs4721888 "GG" genotypic variant (Table 4). These results exhibited the variety of *MACC1* SNPs in HCC progression with consideration of risk factors such as cigarette smoking and alcohol drinking. The *MACC1* SNP rs1990172 is located in an intronic region of the *MACC1* gene 27. Some studies have associated the *MACC1* SNP rs1990172 with poor prognosis and worse survival. However, the exact role of rs1990172 in different cancers remained controversial and inconsistency. In colorectal cancer, alcohol and smoking were suggested as risk factors of premalignant and malignant colorectal neoplasms 35. Moreover, smoking and passive smoking was suggested as a risk factor for pulmonary metastasis of colorectal cancer and be associated with an increased risk of colorectal cancer, respectively 36, 37. Horvat et al. revealed that in Slovenian population, patients with TT genotype of SNP rs1990172 in gene *MACC1* were associated with worse disease-free survival (DFS), and patients with T3/T4 staging were found to have higher frequency of *MACC1* SNP rs1990172 TT genotype 26. However, a study reported by Lang et al. has demonstrated that carriers of the G-allele of SNP rs1990172 showed a significantly decreased overall survival in Austrian colorectal cancer patients 22. In breast cancer, Muendlein et al. reported that in 164 consecutive white patients with HER2-positive breast cancer, carriers of the G-allele of *MACC1* SNP rs1990172 showed increased risk for progression or death to event-free survival and overall survival after age and tumour stage was adjusted 24. In contrast, Dai et al. reported that no relationships was found between rs1990172 and breast cancer risk, whereas the CTGG and CTCG haplotypes of rs975263, rs1990172, rs3735615, and rs4721888 were significantly associated with decreased susceptibility to breast cancer 25. Although we did not perform the haplotype analysis in our current study, and the interaction between *MACC1* and carcinogenic risk factors such as smoking and alcohol drinking are not well-understood, it could be proposed that the haplotype of *MACC1* SNPs may play a crucial role in cancer progression since the *MACC1* SNP rs1990172 was observed to exhibit controversial role in different cancers and ethnicities 22, 24-26. On the other hand, for *MACC1* SNP rs4721888, the frequency of rs4721888 GC and GC+CC variants was higher compared with the rs4721888 CC genotype in breast cancer patients, suggesting that the rs4721888 polymorphisms in MACC1 is associated with the risk of breast cancer susceptibility 25. In contrast, no significant association was found between MACC1 rs4721888 and colorectal cancer patients 23 or HCC patients who received liver transplantation 27, respectively. Besides, the previous studies have suggested that *MACC1* is more frequently expressed in vascular invasive HCC and may serve as a marker for HCC prognosis prediction 16, 38. Compared with this result, our study have found that the *MACC1* rs4721888 is associated with higher risk of vascular invasion in HCC patients among drinkers (Table 4), and most of the HCC patients involved in our study have liver cirrhosis (82.3%; Table 1) and classified as Child-Pugh grade A (79.9%; Table 1). Since high intratumoral MACC1 expression was suggested to predict poor outcomes of cryoablation therapy for patients with advanced HCC and Child-pugh class A or B cirrhosis 39, and MACC1 may serve as a marker to predict prognosis in vascular invasive HCC 16. Perhaps the expression of *MACC1* SNP rs4721888 may provide an explanation to these phenomenon, and may serve as marker in early stage of liver cirrhosis and to predict the prognosis of vascular invasive HCC. However, the exact role of *MACC1* SNPs and haplotype in various cancers including HCC remained controversial and incompletely.

AST/ALT ratio was described as a characteristic of acute viral hepatitis or alcoholic hepatitis 40, 41. In chronic viral hepatic illnesses including chronic viral hepatitis, chronic alcoholism, and non-alcoholic fatty liver disease, an elevated AST/ALT ratio could be interpreted as a prediction to evaluate long terms complications such as fibrosis and cirrhosis 40. About 36.2% HCC patients enrolled in our study drank alcohol (Table 1), and some studies have associated the *MACC1* rs975263 polymorphisms with reduced survival and tumor recurrence 23, 25, 27. In the current study, after we examined the association of *MACC1* genotypic frequencies with the HCC laboratory findings, a statistical significant association was found between the *MACC1* rs975263 "AG+GG" variants and aberrant AST/ALT ratio before adjustment of age and alcohol drinking (*p* = 0.021; table 5). This result revealed that the direct impact of rs975263 to aberrant AST/ALT ratio is limited without consideration of risk factors (confounders) including age and alcohol drinking in HCC patients, suggesting a possible synergistic effect of* MACC1* rs4721888 and rs975263 polymorphic variants to HCC poor prognosis in HCC patients among drinkers (Table 4, 5). However, their detail effects and mechanisms required future well-designed study to elucidate it.

In conclusion, our study demonstrated the associations of *MACC1* SNPs to HCC. The *MACC1* rs1990172 "CA + AA" polymorphic variants are associated with lower risk of clinical stage, tumor size, and vascular invasion in HCC patients among smokers. In HCC patients among drinkers, patients with *MACC1* rs4721888 "GC + CC" polymorphisms are associated with higher risk of vascular invasion. The *MACC1* rs975263 polymorphisms is associated with aberrant AST/ALT ratio before adjusted with age and alcohol drinking, and may have a potential synergistic effect with *MACC1* rs4721888 polymorphisms in HCC patients who drink alcohol. The *MACC1* polymorphisms may be applied as a marker or predictor to evaluate HCC progression and prognosis.

## Figures and Tables

**Table 1:** The distributions of demographical characteristics in 1199 controls and 378 patients with HCC.

Variable	Controls (N=1199)	Patients (N=378)	p value
Age (yrs)	Mean ± S.D.	Mean ± S.D.	
	59.4 ± 7.1	63.0 ± 11.3	p < 0.001*
Gender			
Male	839 (70.0%)	266 (70.4%)	
Female	360 (30.0%)	112 (29.6%)	p = 0.884
Cigarette smoking			
No	728 (60.7%)	227 (60.0%)	
Yes	471 (39.3%)	151 (40.0%)	p = 0.818
Alcohol drinking			
No	1030 (85.9%)	241 (63.8%)	
Yes	169 (14.1%)	137 (36.2%)	p < 0.001*
HBsAg			
Negative		218 (57.7%)	
Positive		160 (42.3%)	
Anti-HCV			
Negative		206 (54.5%)	
Positive		172 (45.5%)	
Stage			
I+II		266 (70.4%)	
III+IV		112 (29.6%)	
Tumor T status			
T1+T2		270 (71.4%)	
T3+T4		108 (28.6%)	
Lymph node status			
N0		367 (97.1%)	
N1+N2+N3		11 (2.9%)	
Metastasis			
M0		360 (95.2%)	
M1		18 (4.8%)	
Child-Pugh grade			
0 and A		302 (79.9%)	
B or C		76 (20.1%)	
Liver cirrhosis			
Negative		67 (17.7%)	
Positive		311 (82.3%)	

Mann-Whitney U test or Fisher's exact test was used between healthy controls and patients with HCC. * p value < 0.05 as statistically significant.

**Table 2:** Genotyping and allele frequency of *MACC1* single nucleotide polymorphism (SNP) in HCC and normal controls.

Variable	Controls (N=1199) (%)	Patients (N=378) (%)	OR (95% CI)	AOR (95% CI)^a^
**rs1990172**				
CC	864 (72.1%)	269 (71.2%)	1.000 (reference)	1.000 (reference)
CA	312 (26.0%)	99 (26.2%)	1.019 (0.782-1.327)	1.046 (0.792-1.382)
AA	23 (1.9%)	10 (2.6%)	1.397 (0.657-2.971)	1.397 (0.631-3.090)
CA+AA	335 (27.9%)	205 (28.8%)	1.045 (0.809-1.350)	1.071 (0.819-1.402)
**rs975263**				
AA	797 (66.4%)	243 (64.3%)	1.000 (reference)	1.000 (reference)
AG	358 (29.9%)	124 (32.8%)	1.136 (0.806-1.344)	1.178 (0.905-1.533)
GG	44 (3.7%)	11 (2.9%)	0.820 (0.417-1.612)	0.838 (0.414-1.695)
AG+GG	402 (33.6%)	135 (35.7%)	1.101 (0.865-1.403)	1.140 (0.883-1.471)
**rs3095007**				
CC	971 (81.0%)	311 (82.3%)	1.000 (reference)	1.000 (reference)
CA	216 (18.0%)	67 (17.7%)	0.968 (0.716-1.310)	0.961 (0.699-1.321)
AA	12 (1.0%)	0 (0.0%)	-	-
CA+AA	228 (19.0%)	67 (17.7%)	0.917 (0.679-1.239)	0.913 (0.666-1.253)
**rs4721888**				
GG	618 (51.6%)	207 (54.8%)	1.000 (reference)	1.000 (reference)
GC	481 (40.1%)	141 (37.3%)	0.875 (0.685-1.118)	0.813 (0.628-1.053)
CC	100 (8.3%)	30 (7.9%)	0.896 (0.578-1.387)	0.839 (0.527-1.336)
GC+CC	581 (48.4%)	171 (45.2%)	0.879 (0.697-1.108)	0.818 (0.640-1.044)
**rs3735615**				
GG	838 (69.9%)	264 (69.8%)	1.000 (reference)	1.000 (reference)
GC	316 (26.4%)	105 (27.8%)	1.055 (0.813-1.369)	1.083 (0.823-1.425)
CC	45 (3.7%)	9 (2.4%)	0.635 (0.307-1.317)	0.575 (0.266-1.245)
GC+CC	361 (30.1%)	114 (30.2%)	1.002 (0.779-1.290)	1.016 (0.780-1.325)

^a^ Adjusted for the effects of age and alcohol drinking.

**Table 3:** Odds ratio (OR) and 95% confidence interval (CI) of clinical status and *MACC1* rs1990172 genotypic frequencies in HCC patients among smokers.

Variable	Genotypic frequencies			
	CC (N=109)	CA+AA (N=42)	OR (95% CI)	p value
Clinical Stage				
Stage I/II	68 (62.4%)	34 (81.0%)	1.00	p=0.032^*^
Stage III/IV	41 (37.6%)	8 (19.0%)	0.390 (0.165-0.924)	
Tumor size				
≦ T2	67 (61.5%)	34 (81.0%)	1.00	p=0.026^*^
> T2	42 (38.5%)	8 (19.0%)	0.375 (0.159-0.888)	
Lymph node metastasis				
No	106 (97.3%)	40 (95.2%)	1.00	p=0.541
Yes	3 (2.7%)	2 (4.8%)	1.767 (0.285-10.967)	
Distant metastasis				
No	104 (95.4%)	40 (95.2%)	1.00	p=0.963
Yes	5 (4.6%)	2 (4.8%)	1.040 (0.194-5.580)	
Vascular invasion				
No	87 (79.8%)	40 (95.2%)	1.00	p=0.034^*^
Yes	22 (20.2%)	2 (4.8%)	0.198 (0.044-0.882)	
Child-Pugh grade				
0 or A	83 (76.2%)	37 (88.1%)	1.00	p=0.111
B or C	26 (23.8%)	5 (11.9%)	0.431 (0.154-1.211)	
HBsAg				
Negative	66 (60.6%)	21 (50.0%)	1.00	p=0.241
Positive	43 (39.4%)	21 (50.0%)	1.535 (0.750-3.142)	
Anti-HCV				
Negative	60 (55.1%)	22 (52.4%)	1.00	p=0.768
Positive	49 (44.9%)	20 (47.6%)	1.113 (0.545-2.273)	
Liver cirrhosis				
Negative	21 (19.3%)	5 (11.9%)	1.00	p=0.288
Positive	88 (80.7%)	37 (88.1%)	1.766 (0.619-5.037)	

The ORs with analyzed by their 95% CIs were estimated by logistic regression models. > T2: multiple tumor more than 5 cm or tumor involving a major branch of the portal or hepatic vein(s) * p value < 0.05 as statistically significant.

**Table 4:** Odds ratio (OR) and 95% confidence interval (CI) of clinical status and *MACC1* rs4721888 genotypic frequencies in HCC patients among drinkers.

Variable	Genotypic frequencies			
	GG (N=69)	GC+CC (N=68)	OR (95% CI)	p value
Clinical Stage				
Stage I/II	53 (76.8%)	45 (66.2%)	1.00	p=0.170
Stage III/IV	16 (23.2%)	23 (33.8%)	1.693 (0.798-3.590)	
Tumor size				
≦ T2	52 (75.4%)	46 (67.6%)	1.00	p=0.318
> T2	17 (24.6%)	22 (32.4%)	1.463 (0.693-3.088)	
Lymph node metastasis				
No	66 (95.6%)	65 (95.6%)	1.00	p=0.985
Yes	3 (4.4%)	3 (4.4%)	1.015 (0.198-5.216)	
Distant metastasis				
No	65 (94.2%)	63 (92.6%)	1.00	p**=**0.714
Yes	4 (5.8%)	5 (7.4%)	1.290 (0.331-5.023)	
Vascular invasion				
No	63 (91.3%)	50 (73.5%)	1.00	p=0.009^*^
Yes	6 (8.7%)	18 (26.5%)	3.780 (1.396-10.230)	
Child-Pugh grade				
0 or A	56 (81.2%)	51 (75.0%)	1.00	p=0.385
B or C	13 (18.8%)	17 (25.0%)	1.436 (0.635-3.246)	
HBsAg				
Negative	41 (59.4%)	40 (58.8%)	1.00	p=0.943
Positive	28 (40.6%)	28 (41.2%)	1.025 (0.519-2.026)	
Anti-HCV				
Negative	37 (53.6%)	38 (55.9%)	1.00	p=0.791
Positive	32 (46.4%)	30 (44.1%)	0.913 (0.466-1.789)	
Liver cirrhosis				
Negative	8 (11.6%)	13 (19.1%)	1.00	p=0.226
Positive	61 (88.4%)	55 (80.9%)	0.555 (0.214-1.439)	

The ORs with analyzed by their 95% CIs were estimated by logistic regression models. > T2: multiple tumor more than 5 cm or tumor involving a major branch of the portal or hepatic vein(s) * *p* value < 0.05 as statistically significant.

**Table 5:** Association of *MACC1* genotypic frequencies with the HCC laboratory findings.

Characteristic	α-Fetoprotein ^a^ (ng/mL)	AST^ a^ (IU/L)	ALT ^a^ (IU/L)	AST/ALT ^a^ ratio
**rs1990172**				
CC	612.4 ± 212.4	49.1 ± 4.3	78.1 ± 32.5	1.23 ± 0.03
CA+AA	848.6 ± 368.7	48.5 ± 5.8	43.7 ± 4.1	1.17 ± 0.02
*p* value	0.579	0.932	0.294	0.130
				
**rs975263**				
AA	652.5 ± 231.1	49.9 ± 4.7	81.5 ± 35.4	1.24 ± 0.03
AG+GG	729.9 ± 305.7	47.1 ± 4.9	43.1 ± 3.5	1.16 ± 0.02
*p* value	0.842	0.686	0.281	0.021
				
**rs3095007**				
CC	677.8 ± 207.6	47.8 ± 3.9	72.9 ± 28.7	1.22 ± 0.02
CA+AA	683.5 ± 399.3	54.0 ± 8.6	49.0 ± 6.0	1.16 ± 0.03
*p* value	0.990	0.487	0.417	0.095
				
**rs4721888**				
GG	550.7 ± 213.0	52.8 ± 5.9	92.7 ± 44.6	1.20 ± 0.02
GC+CC	819.5 ± 308.5	44.7 ± 3.5	41.8 ± 2.9	1.22 ± 0.03
*p* value	0.474	0.236	0.256	0.511
				
**rs3735615**				
GG	763.9 ± 241.2	49.8 ± 4.5	79.5 ± 33.4	1.23 ± 0.03
GC+CC	481.6 ± 249.5	46.9 ± 5.5	42.7 ± 3.8	1.16 ± 0.02
*p* value	0.416	0.677	0.276	0.055

Mann-Whitney U test was used between two groups. ^a^ Mean ± S.E.
